# HMGA1 is a novel transcriptional regulator of the *FoxO1* gene

**DOI:** 10.1007/s12020-017-1445-8

**Published:** 2017-10-19

**Authors:** Biagio Arcidiacono, Eusebio Chiefari, Sebastiano Messineo, Francesco L. Bilotta, Ida Pastore, Domenica M. Corigliano, Daniela P. Foti, Antonio Brunetti

**Affiliations:** Department of Health Sciences, University “Magna Græcia” of Catanzaro, Viale Europa (Località Germaneto), 88100 Catanzaro, Italy

**Keywords:** HMGA1, FoxO1, Insulin signaling, Gene transcription, DNA/chromatin interaction

## Abstract

**Purpose:**

The forkhead transcription factor (FoxO1) is a master transcriptional regulator of fundamental cellular processes ranging from cell proliferation and differentiation to inflammation and metabolism. However, despite its relevance, the mechanism(s) underlying *FoxO1* gene regulation are largely unknown. We have previously shown that the chromatin factor high-mobility group A1 (HMGA1) plays a key role in the transcriptional regulation of glucose-responsive genes, including some that are involved in FoxO1-mediated glucose metabolism. Here we investigated the impact of HMGA1 on *FoxO1* gene expression.

**Methods:**

FoxO1 protein and gene expression studies were performed by Western blot analysis combined with qRT-PCR of material from human cultured cells and EBV-transformed lymphoblasts, and from primary cultured hepatocytes from wild-type and *Hmga1* ^–/–^ mice. Reporter gene assays and chromatin immunoprecipitation for binding of HMGA1 to the endogenous *FoxoO1* locus were performed in cells overexpressing HMGA1 and in cells pretreated with siRNA targeting HMGA1.

**Results:**

HMGA1 increased FoxO1 mRNA and protein expression in vitro, in cultured HepG2 and HEK-293 cells by binding *FoxO1* gene promoter, thereby activating *FoxO1* gene transcription. Forced expression of HMGA1 in primary cultured hepatocytes from *Hmga1* ^–/–^ mice and in EBV-transformed lymphoblasts from subjects with reduced expression of endogenous HMGA1 increased FoxO1 mRNA and protein levels.

**Conclusion:**

These findings may contribute to the understanding of *FoxO1* gene regulation and its role in metabolism.

## Introduction

Forkhead box protein O1 (FoxO1), a member of the forkead family of transcription factors characterized by a highly conserved forkhead DNA-binding domain, regulates the transcription of genes involved in a wide spectrum of fundamental biological processes, like cell proliferation and differentiation, apoptosis and DNA repair, inflamation and stress response, insulin sensitivity and energy metabolism [1–4]. As a crucial effector of insulin action, FoxO1 is highly expressed in insulin-responsive tissues, including pancreas and liver, skeletal muscle, white and brown adipose tissue, skeleton and brain, at which levels FoxO1 exerts effects over glucose metabolism, insulin sensitivity and energy expenditure [5–9]. In particular, in the liver, FoxO1 stimulates gluconeogenic genes, such as phosphoenolpyruvate carboxykinase (*PEPCK*) and glucose-6-phosphatase (*G6Pase)*, as well as genes involved in glucose counterregulation, including the insulin-like growth factor binding protein 1 (*IGFBP1*) gene [10, 11], thus ensuring fasting euglycemia. After a meal, by triggering the phosphorylation of FoxO1 via AKT, insulin induces its detachment from DNA and its nuclear export, thereby inhibiting gluconeogenesis and suppressing hepatic glucose output [12–15]. Consequently, under insulin resistance conditions, FoxO1 phosphorylation is blunted, and the transcriptional activation of gluconeogenic genes is abnormally sustained, which confers increased susceptibilty to type 2 diabetes (T2D) mellitus [16].

While insulin regulation of FoxO1 protein is fairly well understood [17, 18] the mechanism(s) regulating *FoxO1* gene expression have been poorly investigated and poorly documented, so that the only data available in this regard refer to the upregulation of *FoxO1* gene in response to E2F-1, a transcription factor involved in cell cycle progression and apoptosis [19], the homeodomain transcription factor ALX3 [20], FoxC1 [21], and FoxO1 itself [22]. Also, it has been reported that *FoxO1* mRNA levels were increased in muscle of fasted or calorically restricted rats [23, 24], supporting the existence of a nutrient deprivation-induced mechanism activating *FoxO1* gene transcription. However, the transcription factors mediating this activation have not been identified.

The high-mobility group A1 (HMGA1) protein is a nuclear architectural factor that belongs to the superfamily of nonhistone chromatin-binding proteins. By binding to AT-rich regions of DNA, HMGA1 can transactivate gene promoters through mechanisms that facilitate the assembly and stability of stereospecific DNA–protein complexes (so-called enhanceosomes), thereby activating or repressing a variety of mammalian genes [25–27]. We previously showed that HMGA1 is an important novel mediator of glucose homeostasis in vivo, playing a crucial role in the transcriptional regulation of a variety of genes actively contributing to the maintenance of blood glucose levels, such as insulin [28] and the insulin receptor genes [27, 29, 30], in addition to a series of insulin-target genes, including the gluconeogenic genes *PEPCK* and *G6Pase* [11], as well as genes encoding proteins that are involved in glucose counterregulation (e.g., IGFBP1 and the retinol-binding protein 4 (RBP4)) [11, 31–33]. A role for HMGA1 as a nuclear target of insulin signaling has been reported by us before, showing that the insulin-mediated postprandial glucose disposal is mediated by detachment of HMGA1 from gluconeogenic gene promoters in a way that resembles the functional association between insulin and FoxO1 [11]. Accordingly, defects in *HMGA1* gene and protein expression have been linked to insulin resistance and T2D in humans and mice [30, 34–38], whereas protection from these metabolic disorders has been reported in transgenic mice that overexpress HMGA1 [39].

Taking into account the above considerations, here we explored the possibility that HMGA1 may be involved in the transcriptional regulation of *FoxO1* gene.

## Materials and methods

### Cell cultures, protein extracts and Western blot

Human embryonic kidney 293 (HEK-293) cells and HepG2 human hepatoma cells were cultured in Dulbecco's modified Eagle medium and RPMI 1640 medium, respectively, supplemented with 10% fetal bovine serum (FBS) (Gibco Laboratories, Grand Island, NY, USA), 2 mM glutamine, penicillin (100 U/ml), and streptomycin (100 µg/ml) in a humidified 5% CO_2_ atmosphere at 37 °C. Cryopreserved primary hepatocytes from HMGA1^–/–^ and wild-type mice [11] were cultured on matrigel-coated six-well plates in Williams E media (Sigma) supplemented with 10% FBS and then utilized in subsequent experiments. Cultured lymphoblast cell lines transformed with Epstein-Barr virus (EBV) were from normal volunteers and previously identified diabetic subjects carrying *HMGA1* gene defects [30, 34, 40], and maintained in RPMI 1640 medium supplemented with 15% FBS. Nuclear protein extracts were prepared as previously [29] and final protein concentration in the extracts was determined by the modified Bradford method (Bio-Rad Laboratories, Hercules, CA, USA). The antibodies used for Western blot studies were: anti-HMGA1 [41], anti-FoxO1 (Santa Cruz Biotechnology, Santa Cruz, CA) and anti Sp1 [42].

### Plasmid construction and transfections

Human *FoxO1* gene promoter region (−1989 to −155 bp relative to the start site of transcription) was amplified from the BAC clone RP11–181D10 (Invitrogen Life Technology Corporation, Carlsbad, Calif. USA), using modified specific primers. After sequence analysis, the resulting product was cloned upstream to the *luciferase* (*Luc*) gene into pGL3 basic vector (Promega, Madison, WI, USA), to obtain the recombinant plasmid *FoxO1*-*Luc*. This construct was transiently transfected into HepG2 or HEK-293 cells, using the LipofectAMINE 2000 reagent (Invitrogen), in the presence or absence of an effector vector (pcDNA3HA-HMGA1a) for the HMGA1a isoform protein, as obtained by subcloning the BamH1/EcoR1 fragment from pcDNA1-HMGI into the pcDNA3HA plasmid (Invitrogen) [27]. Luc activity was assayed 48 h later in a luminometer (Turner Biosystems Inc., CA, USA), using the dual-luciferase reporter assay system (Promega, Madison, Wis., USA). For gene silencing experiments, cells at 40–50% confluency were transfected with 100–200 pmol anti-*HMGA1* siRNA plus nonspecific control siRNA with a similar GC content (Santa Cruz Biotechnology, Santa Cruz, CA) and maintained in culture for 72 h, after which cells were removed from the culture plates and subjected to a second transfection with the same siRNAs for an additional 72 h. Renilla control vector served as an internal control of transfection efficiency. EBV-transformed lymphoblasts were transfected with expression vector containing the HMGA1 cDNA as described previously [27]. For the selection of transfected cells, lymphoblasts were cotransfected with the pEGFP vector (Clontech) and collected 18 h later with a FACS Vantage cell sorter (Becton Dickinson). Sorted cells were maintained in culture and used for gene and protein expression analyses 72 h later [26].

### Chromatin immunoprecipitation (ChIP)

ChIP was performed in HepG2 and EBV-transformed lymphoblasts, either untreated or pre-treated with *HMGA1* siRNA or with *HMGA1* cDNA, as described previously [11, 30, 43]. Formaldehyde-fixed DNA–protein complex was immunoprecipitated with anti-HMGA1 antibody [30, 44], and the following primers for the *FoxO1* gene promoter were used for PCR amplification of ChIP-ed DNA (30 cycles), using PCR ready-to-go beads (Amersham Pharmacia Biotech): human *FoxO1* (NT_007819) for 5′-CCCAAGGCTTTGGTCCTATC-3′, rev 5′- GCCGGATTCACTGTATTCTTG -3′. PCR products were electrophoretically resolved on 1.5% agarose gel and visualized by ethidium bromide staining.

### Real-time PCR

For qRT-PCR, total cellular RNA was extracted from cells using the RNAqueous-4PCR kit and subjected to DNase treatment (Ambion). RNA levels were normalized against 18 S ribosomal RNA in each sample, and cDNAs were synthesized from 2 μg of total RNA using the RETROscript first strand synthesis kit (Ambion). Primers for human *FoxO1* (NM_002015.3) (5′- AAGGATAAGGGTGACAGCAACAG-3′; 5′- TTGCTGTGTAGGGACAGATTATGAC-3′), and mouse *FoxO1* (NM_019739) (5′- CAAAGTACACATACGGCCAATCC-3′; 5′- CGTAACTTGATTTGCTGTCCTGAA-3′), were designed according to sequences from the GenBank database. qRT-PCR was performed as previously described [32], using RPS9 cDNA as an internal standard. All PCR reactions were done in triplicates.

### Statistics

Statistical significance was evaluated by Student’s *t* test. *P* < 0.05 was considered significant. All bar graph data shown are mean ± S.E.M.

## Results

### HMGA1 induces *FoxO1* gene transcription

**Fig. 1:**
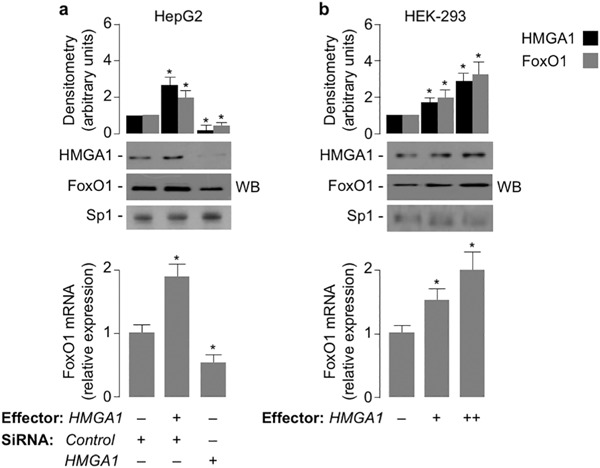
*FoxO1* gene expression is induced by HMGA1. **a** pcDNA3-*HMGA1* effector vector was transfected into HepG2 cells, either in the absence or presence of siRNA against HMGA1, or a non-targeting control siRNA, and endogenous *FoxO1* mRNA expression was measured thereafter. **b** Increasing amounts (0, 0.5, 1 µg) of pcDNA3-*HMGA1* effector vector were transfected into HEK-293 cells, and *FoxO1* mRNA was measured as in **a**. Results are means ± S.E.M. of three independent experiments, each in triplicate. *P < 0.05 vs. control (first column in each condition). Representative Western blots (WB) of FoxO1 and HMGA1 out of three independent experiments for each condition are shown in the autoradiograms. Bar graphs above the gel panels are derived from densitometric scanning of WBs, using the ImageJ software program. *P < 0.05 vs. controls (first columns in each condition). Sp1, control of nuclear protein loading

**Fig. 2:**
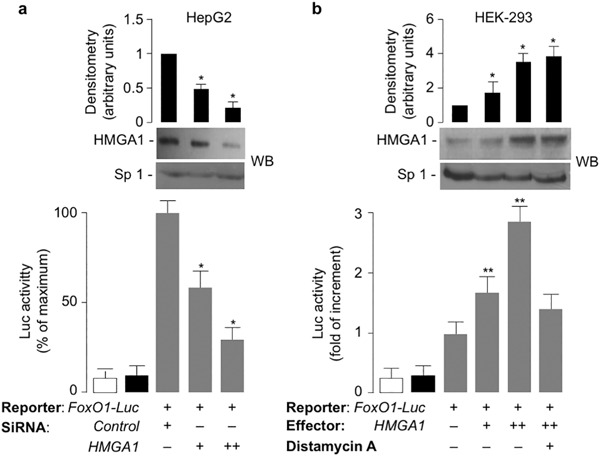
HMGA1 regulates *FoxO1* gene transcription. **a** *FoxO1*-Luc reporter vector was transfected into HepG2 cells, either in the absence or presence of siRNA against HMGA1, or a nontargeting control siRNA, and Luc activity was measured 72 to 96 h later. **b** *FoxO1*-Luc reporter vector was transfected into HEK-293 cells, with increasing amounts (0, 0.5, 1 µg) of pcDNA3-*HMGA1* effector vector, in the absence or presence of 10 µM distamycin A, and Luc activity was measured as in **a**. In both experimental conditions, Luc activity from the reporter plasmid was normalized by the renilla Luc activity produced from a pRL Renilla-Luc control vector cotransfected as an internal control. White bar, mock (no DNA); black bar, (pGL3 basic vector, without the *FoxO1* promoter). Results are means ± S.E.M. of three independent experiments, each in triplicate. *P < 0.05 compared with nontargeting (control) siRNA; **P < 0.05 vs. the *FoxO1-Luc*, in the absence of a HMGA1 effector vector, which is assigned an arbitrary value of 1. Representative WBs of HMGA1 out of three independent experiments for each condition are shown in the autoradiograms. Bar graphs above the gel panels are derived from densitometric scanning of WBs, using the ImageJ software program. *P < 0.05 vs. control (first column in each condition). Sp1, control of nuclear protein loading

To explore the possibility that HMGA1 could be involved in the regulation of FoxO1 expression, we first carried out in vitro studies in HepG2 and HEK-293 cells, two human cell lines which express high and low levels of endogenous HMGA1, respectively. Following transient transfections with either HMGA1 expression vector or siRNA targeting *HMGA1*, both *FoxO1* mRNA and protein levels were increased in HepG2 cells overexpressing HMGA1, and were reduced in cells pretreated with siRNA targeting *HMGA1* (Fig. 1a). Overexpression of increasing amounts of HMGA1 in HEK-293 cells increased *FoxO1* mRNA and protein levels in a dose-dependent manner (Fig. 1b). To test the hypothesis that HMGA1 could play a role in the transcriptional regulation of the *FoxO1* gene, we performed reporter gene analysis in HepG2 and HEK-293 cells, after transfecting both cell lines with the *FoxO1*-*Luc* reporter plasmid bearing the human *FoxO1* promoter sequence upstream to the *luciferase* reporter gene, in the presence of increasing amounts of either siRNA targeting *HMGA1* or HMGA1 expression vector, respectively. As shown in Fig. 2a, *FoxO1*-*Luc* activity was reduced in HepG2 cells pretreated with siRNA targeting *HMGA1*, whereas overexpression of HMGA1 in HEK-293 cells increased *FoxO1*-*Luc* activity in a dose-dependent manner and this effect was reduced in cells treated with distamycin A, a small molecule inhibitor of HMGA1 protein binding to DNA [11] (Fig. 2b), thus indicating that HMGA1 is required for the regulation of this promoter in vitro.

### HMGA1 binds *FoxO1* gene promoter

**Fig. 3:**
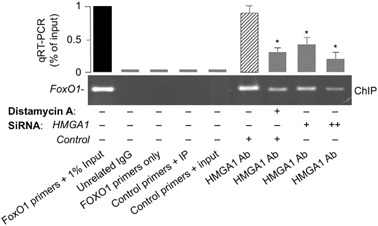
HMGA1 binds *FoxO1* promoter in living cells. ChIP of the *FoxO1* promoter gene in HepG2 cells untreated or pretreated with either distamycin A or HMGA1 siRNA. ChIP was carried out using an anti-HMGA1 specific antibody (Ab). qRT-PCRs of ChIP-ed samples are shown in each condition. A representative ChIP assay out of at least three independent experiments is presented. *P < 0.05 vs. nontargeting control siRNA (slashed bar)

Based on the above functional observations and the presence of several putative binding sites for HMGA1 in the promoter region of *FoxO1*, as revealed by sequence analysis with MatInspector software (version 8.1, Genomatix, http://www.genomatix.de/), we performed ChIP coupled with qRT-PCR of ChIP-ed samples, showing that binding of HMGA1 to the endogenous *FoxO1* chromosomal locus was manifest in living HepG2 cells naturally expressing HMGA1, and was decreased in cells that were exposed to distamycin A (Fig. 3). The reduction in HMGA1 occupancy at the endogenous *FoxO1* locus was also reduced by treating cells with siRNA against HMGA1 (Fig. 3), further substantiating the functional role of HMGA1 at the level of *FoxO1* gene promoter. Thus, taken together, these data demonstrate that HMGA1 is of major importance for the transcriptional regulation of the *FoxO1* gene, suggesting that functional impairment of HMGA1 may contribute to reduced expression of FoxO1, thereby to the inhibition of FoxO1-mediated gene transcription and function (e.g., hepatic gluconeogenesis).

### *FoxO1* gene expression in primary cultured hepatocytes from *Hmga1*^*–/–*^ mice

**Fig. 4:**
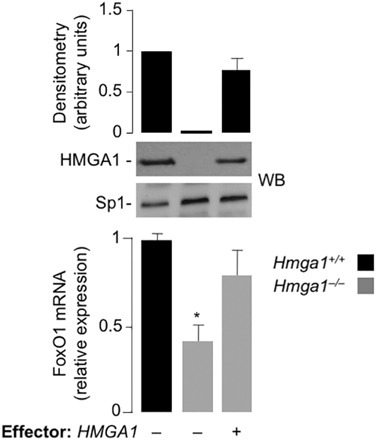
*FoxO1* gene expression in primary culture cells*. FoxO1* mRNA abundance was measured in primary cultured hepatocytes from both *Hmga1* ^+/+^ (black bar) and *Hmga1* ^–/–^ (gray bar) mice, before and after transfection of the cells with the *pcDNA3-HMGA1* expression vector. Results are means ± S.E.M. of five independent experiments. *P < 0.05 vs. *Hmga1* ^+/+^ hepatocytes. Representative WBs are shown in each condition. Bar graph above the gel panel is derived from densitometric scanning of anti-HMGA1 WB, using the ImageJ software program. Sp1, control of nuclear protein loading

We previously showed that HMGA1 plays an essential role in the transcriptional activation of the FoxO1-dependent gluconeogenic genes *PEPCK* and *G6Pase*, as well as on the transcription of the insulin-target gene *IGFBP1*, thereby contributing to the maintenance of fasting euglycemia [11, 30]. Also, consistently with our findings above and the role of FoxO1 in hepatic gluconeogenesis, plasma glucose concentration in *Hmga1* ^***–/–***^ mice was lower than in wild-type animals [32], suggesting that HMGA1 may be required for *FoxO1* gene expression and function also in vivo, in whole animal. This hypothesis has now been further supported in the present work, by additional studies of primary cultured hepatocytes from *Hmga1* ^–/–^ and wild-type mice, showing that the mRNA expression of FoxO1 was lower in *Hmga1* ^–/–^ hepatocytes compared to wild-type hepatocytes (Fig. 4). Restoration of HMGA1 expression following transfection of primary cultured hepatocytes with a HMGA1 expression vector, enhanced mRNA levels for FoxO1, thus further proving that HMGA1 is essential for *FoxO1* gene expression.

### FoxO1 is reduced in subjects carrying defects in HMGA1

**Fig. 5:**
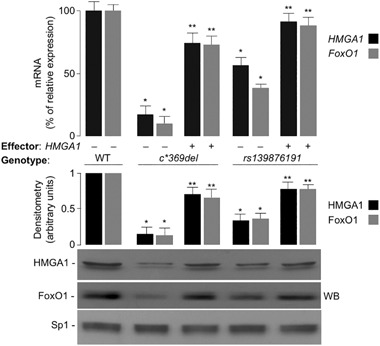
*FoxO1* expression in EBV-transformed lymphoblasts from subjects expressing variable amounts of HMGA1. mRNA and protein expression (WB) for both HMGA1 and FoxO1 were concomitantly measured in cultured EBV-transformed lymphoblasts from normal subjects (WT, wild-type, *n* = 6) and subjects carrying *HMGA1* gene variants (c.*369del and *rs139876191*; *n* = 3 and 4, respectively) that decrease HMGA1 protein expression [30, 34], either in the absence (−) or presence (+) of the effector vector for HMGA1. qRT-PCR of HMGA1 and FoxO1 mRNA levels are shown as percent of maximal value (WT, 100%). Results are means ± S.E.M. for three separate assays. *P < 0.05 vs. controls (WT). **P < 0.05 vs. untransfected cells in each variant group. Representative WBs for HMGA1 and FoxO1 are shown. Bar graphs above the gel panels are derived from densitometric scanning of WBs, using the ImageJ software program. *P < 0.05 vs. controls (WT); **P < 0.05 vs. untransfected cells in each variant group. Sp1, control of nuclear protein loading

**Fig. 6:**
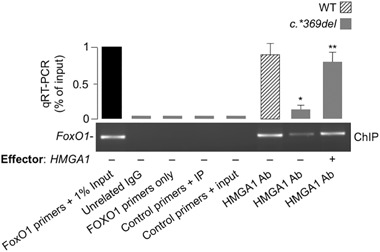
Binding of HMGA1 to *FoxO1* promoter in subjects’ cells. The occupancy of the *FoxO1* gene promoter by HMGA1 was measured by ChIP in EBV-transformed lymphoblasts from normal WT subjects (slashed bar, *n* = 3) and subjects carrying the *HMGA1* c.*369del variant (gray bars, *n* = 3), using an anti-HMGA1 specific antibody (Ab), either before or after transfecting cells with the c.*369del variant with an HMGA1 effector vector. qRT-PCRs of ChIP-ed samples are shown in each condition. *P < 0.05 vs. WT subjects; **P < 0.05 vs. untransfected cells carrying the *HMGA1* c.*369del variant. A representative ChIP assay is presented

In the light of the above experimental results, we thought to investigate the expression of FoxO1 in individuals with variable amounts of HMGA1. To this end, FoxO1 expression levels were measured in cultured EBV-transformed lymphoblasts from previously selected individuals with and without insulin resistance and T2D, with and without *HMGA1* gene defects adversely affecting HMGA1 protein production [30, 34–36]. As shown in Fig. 5, *FoxO1* mRNA abundance was reduced in cells expressing low levels of HMGA1, and this reduction paralleled the decrease in FoxO1 protein expression, as detected by immunoblots from nuclear extracts of cultured lymphoblasts. Restoration of HMGA1 protein expression also in these subjects’ cells enhanced *FoxO1* expression (Fig. 5), thus indicating that, as observed in murine primary hepatocytes, HMGA1 can also regulate *FoxO1* expression in human-derived cells. The role of HMGA1 on *FoxO1* promoter, in humans, was corroborated by ChIP experiments coupled with qRT-PCR of ChIP-ed DNA, showing that binding of HMGA1 to the endogenous *FoxO1* chromosomal locus was low in living EBV-transformed cells from subjects with reduced expression of HMGA1, and was considerably increased in EBV cells after transfection of the expression vector for HMGA1 (Fig. 6).

## Discussion

We previously provided data indicating that HMGA1 may play a fundamental role in insulin signaling and the transcriptional regulation of glucose metabolism [11, 30–32]. This notion is based mainly on the observation that HMGA1 is a key regulator of a variety of insulin-target genes, including the insulin receptor gene, as well as the gluconeogenic genes *PEPCK* and *G6Pase*, in addition to other genes encoding for proteins important in glucose counterregulation and homeostasis such as, *IGFBP1* and *RBP4* [11, 31, 32].

On the other hand, the specific action of the transcription factor FoxO1 in regulating glucose homeostasis has been reported in several studies, which have also contributed significantly to our understanding of the mechanism of action and the physiological significance of FoxO1 in this context. For example, cytoplasm retention of FoxO1 via insulin-induced phosphorylation is suggested to be a mechanism of insulin-mediated gene repression, thus a critical regulator of peripheral insulin action [45–47]. The possibility that HMGA1, by affecting *Foxo1* gene and protein expression can be a component of this regulation, would constitute an interesting finding of the present work, which, however, deserves further research to be better assessed. Compared to previous investigations, for the first time in the present study, we report the identification of HMGA1 as a novel nuclear modulator of the *Foxo1* gene, which might play a major role in FoxO1-mediated transactivation in mammals.

In our study, the involvement of HMGA1 in the expression of FoxO1 is underlined by the following facts: first, HMGA1 enhances *FoxO1* gene expression and promoter activity in cultured cells; second, binding of HMGA1 to the endogenous *FoxO1* chromosomal locus is increased in living cells naturally expressing HMGA1, and is reduced in cells exposed either to distamycin A or siRNA against HMGA1; third, FoxO1 abundance is low in both primary cultured hepatocytes from *Hmga1* ^–/–^ mice and EBV-transformed lymphoblasts from subjects with reduced expression of HMGA1, in which restoration of HMGA1 protein production enhances *FoxO1* gene and protein expression. Altogether, these observations strengthen the role of HMGA1 as a novel transcriptional activator of *FoxO1* gene, lending support to the view that HMGA1, which is upregulated via the cAMP-signaling pathway during fast [32, 33], may play a pivotal role in maintaining fasting euglycemia, in vivo. Also, as concomitant insulin resistance and insulin hypersensitivity in peripheral tissues may paradoxically coexist in certain adverse metabolic conditions in which insulin action is precluded [30, 32], it is tempting to hypothesize that the reduction of HMGA1, by adversely affecting FoxO1 expression, might reflect an adaptive mechanism to increase insulin action.

From a clinical point of view, the observation that diabetic subjects with *HMGA1* gene defects might display also a decrease in FoxO1 protein expression supports the notion that patients with T2D who have these defects may have a different clinical course than other patients with T2D. In this respect, we recently reported that diabetic patients carrying the *rs139876191 HMGA1* variant (which causes reduced HMGA1 protein expression) had a significantly lower risk of proliferative diabetic retinopathy, compared to non-carrier patients, and this protective effect was attributed, at least in part, to downregulation of the vascular endothelial growth factor A, a major activator of neovascularization [48]. In this context, the involvement of FoxO1 in the induction of genes involved in diabetic retinopathy and its activation during the early inflammatory phase of diabetic retinopathy has been reported [49, 50]. Thus, the protective effect of the *HMGA1 rs139876191* variant on diabetic retinopathy may also be mediated through a process that involves inactivation of *FoxO1* gene and protein expression. Although the relevance of our data to the clinical setting requires further investigation, they provide compelling evidence for the identification of HMGA1 as a novel nuclear activator of *FoxO1* gene transcription, which may help explaining noted differences in diabetes phenotypes.

In our opinion, these findings offer new biological and mechanistic insights into the mechanism(s) underlying *FoxO1* gene regulation and might be useful in understanding the molecular basis of clinical phenotypes in those conditions where insulin action is compromised (e.g., T2D and other insulin-resistant states). Understanding these mechanisms could help in identifying novel therapeutic targets for preventing and treating these diseases.

## References

[1] Monsalve M, Olmos Y (2011). The complex biology of FOXO. Curr. Drug Targets.

[2] Accili D, Arden KC (2004). FoxOs at the crossroads of cellular metabolism, differentiation, and transformation. Cell.

[3] Hribal ML, Nakae J, Kitamura T, Shutter JR, Accili D (2003). Regulation of insulin-like growth factor-dependent myoblast differentiation by Foxo forkhead transcription factors. J. Cell Biol..

[4] Nakae J, Kitamura T, Kitamura Y, Biggs WH, Arden KC, Accili D (2003). The forkhead transcription factor Foxo1 regulates adipocyte differentiation. Dev. Cell.

[5] Kousteni S (2012). FoxO1, the transcriptional chief of staff of energy metabolism. Bone.

[6] Puigserver P, Rhee J, Donovan J, Walkey CJ, Yoon JC, Oriente F, Kitamura Y, Altomonte J, Dong H, Accili D, Spiegelman BM (2003). Insulin-regulated hepatic gluconeogenesis through FOXO1-PGC-1alpha interaction. Nature.

[7] Kim MS, Pak YK, Jang PG, Namkoong C, Choi YS, Won JC, Kim KS, Kim SW, Kim HS, Park JY, Kim YB, Lee KU (2006). Role of hypothalamic Foxo1 in the regulation of food intake and energy homeostasis. Nat. Neurosci..

[8] Kitamura YI, Kitamura T, Kruse JP, Raum JC, Stein R, Gu W, Accili D (2005). FoxO1 protects against pancreatic beta cell failure through NeuroD and MafA induction. Cell Metab..

[9] Matsumoto M, Pocai A, Rossetti L, Depinho RA, Accili D (2007). Impaired regulation of hepatic glucose production in mice lacking the forkhead transcription factor foxo1 in liver. Cell. Metab..

[10] Lee PDK, Conover CA, Powell DR (1993). Regulation and function of insulinlike growth factor-binding protein-1. Proc. Soc. Exp. Biol. Med..

[11] Chiefari E, Nevolo MT, Arcidiacono B, Maurizio E, Nocera A, Iiritano S, Sgarra R, Possidente K, Palmieri C, Paonessa F, Brunetti G, Manfioletti G, Foti D, Brunetti A (2012). HMGA1 is a novel downstream nuclear target of the insulin receptor signaling pathway. Sci. Rep..

[12] Biggs WH, Meisenhelder J, Hunter T, Cavenee WK, Arden KC (1999). Protein kinase B/Akt-mediated phosphorylation promotes nuclear exclusion of the winged helix transcription factor FKHR1. Proc. Natl. Acad. Sci. USA.

[13] Matsuzaki H, Daitoku H, Hatta M, Tanaka K, Fukamizu A (2003). Insulin-induced phosphorylation of FKHR (FOXO1) targets to proteosomal degradation. Proc. Natl. Acad. Sci. USA.

[14] Rena G, Prescott AR, Guo S, Cohen P, Unterman TG (2001). Roles of the forkhead in rhabdomyosarcoma (FKHR) phosphorylation sites in regulating 14-3-3 binding, transactivation and nuclear targeting. Biochem. J..

[15] Zhang X, Gan L, Pan H, Guo S, He X, Olson ST, Mesecar A, Adam S, Unterman TG (2002). Phosphorylation of serine 256 suppresses transactivation by FKHR (FOXO1) by multiple mechanisms. Direct and indirect effects on nuclear/cytoplasmic shuttling and DNA binding. J. Biol. Chem..

[16] Pajvani UB, Accili D (2015). The new biology of diabetes. Diabetologia.

[17] Dong XC, Copps KD, Guo S, Li Y, Kollipara R, DePinho RA, White MF (2008). Inactivation of hepatic Foxo1 by insulin signaling is required for adaptive nutrient homeostasis and endocrine growth regulation. Cell Metab..

[18] Samuel VT, Choi CS, Phillips TG, Romanelli AJ, Geisler JG, Bhanot S, McKay R, Monia B, Shutter JR, Lindberg RA, Shulman GI, Veniant MM (2006). Targeting foxo1 in mice using antisense oligonucleotide improves hepatic and peripheral insulin action. Diabetes.

[19] Nowak K, Killmer K, Gessner C, Lutz W (2007). E2F-1 regulates expression of FOXO1 and FOXO3a. Biochim. Biophys. Acta.

[20] García-Sanz P, Mirasierra M, Moratalla R, Vallejo M (2017). Embryonic defence mechanisms against glucose-dependent oxidative stress require enhanced expression of Alx3 to prevent malformations during diabetic pregnancy. Sci. Rep..

[21] Berry FB, Skarie JM, Mirzayans F, Fortin Y, Hudson TJ, Raymond V, Link BA, Walter MA (2008). FOXC1 is required for cell viability and resistance to oxidative stress in the eye through the transcriptional regulation of FOXO1A. Hum. Mol. Genet..

[22] Essaghir A, Dif N, Marbehant CY, Coffer PJ, Demoulin JB (2009). The transcription of FOXO genes is stimulated by FOXO3 and repressed by growth factors. J. Biol. Chem..

[23] Furuyama T, Yamashita H, Kitayama K, Higami Y, Shimokawa I, Mori N (2002). Effects of aging and caloric restriction on the gene expression of Foxo1, 3, and 4 (FKHR, FKHRL1, and AFX) in the rat skeletal muscles. Microsc. Res. Tech..

[24] Imae M, Fu Z, Yoshida A, Noguchi T, Kato H (2003). Nutritional and hormonal factors control the gene expression of FoxOs, the mammalian homologues of DAF-16. J. Mol. Endocrinol..

[25] Reeves R (2001). Molecular biology of HMGA proteins: hubs of nuclear function. Gene.

[26] Sgarra R, Zammitti S, Lo Sardo A, Maurizio E, Arnoldo L, Pegoraro S, Giancotti V, Manfioletti G (2010). HMGA molecular network: from transcriptional regulation to chromatin remodeling. Biochim. Biophys. Acta.

[27] Foti D, Iuliano R, Chiefari E, Brunetti A (2003). A nucleoprotein complex containing Sp1, C/EBPβ, and HMGI-Y controls human insulin receptor gene transcription. Mol. Cell Biol..

[28] Arcidiacono B, Iiritano S, Chiefari E, Brunetti FS, Gu G, Foti DP, Brunetti A (2015). Cooperation between HMGA1, PDX-1 and MafA is essential for glucose-induced insulin transcription in pancreatic beta cells. Front. Endocrinol..

[29] Brunetti A, Manfioletti G, Chiefari E, Goldfine ID, Foti D (2001). Transcriptional regulation of human insulin receptor gene by the high-mobility group protein HMGI(Y). FASEB J..

[30] Foti D, Chiefari E, Fedele M, Iuliano R, Brunetti L, Paonessa F, Manfioletti G, Barbetti F, Brunetti A, Croce CM, Fusco A, Brunetti A (2005). Lack of the architectural factor HMGA1 causes insulin resistance and diabetes in humans and mice. Nat. Med..

[31] Iiritano S, Chiefari E, Ventura V, Arcidiacono B, Possidente K, Nocera A, Nevolo MT, Fedele M, Greco A, Greco M, Brunetti G, Fusco A, Foti D, Brunetti A (2012). The HMGA1-IGF-I/IGFBP system: a novel pathway for modulating glucose uptake. Mol. Endocrinol..

[32] Chiefari E, Paonessa F, Iiritano S, Le Pera I, Palmieri D, Brunetti G, Lupo A, Colantuoni V, Foti D, Gulletta E, De Sarro G, Fusco A, Brunetti A (2009). The cAMP-HMGA1-RBP4 system: a novel biochemical pathway for modulating glucose homeostasis. BMC Biol..

[33] Bianconcini A, Lupo A, Capone S, Quadro L, Monti M, Zurlo D, Fucci A, Sabatino L, Brunetti A, Chiefari E, Gottesman ME, Blaner WS, Colantuoni V (2009). Transcriptional activity of the murine retinol-binding protein gene is regulated by a multiprotein complex containing HMGA1, p54 nrb/NonO, protein-associated splicing factor (PSF) and steroidogenic factor 1 (SF1)/liver receptor homologue 1 (LRH-1). Int. J. Biochem. Cell Biol..

[34] Chiefari E, Tanyolaç S, Paonessa F, Pullinger CR, Capula C, Iiritano S, Mazza T, Forlin M, Fusco A, Durlach V, Durlach A, Malloy MJ, Kane JP, Heiner SW, Filocamo M, Foti DP, Goldfine ID, Brunetti A (2011). Functional variants of the HMGA1 gene and type 2 diabetes mellitus. JAMA.

[35] Pullinger CR, Goldfine ID, Tanyolaç S, Movsesyan I, Faynboym M, Durlach V, Chiefari E, Foti DP, Frost PH, Malloy MJ, Brunetti A, Kane JP (2014). Evidence that an HMGA1 gene variant associates with type 2 diabetes, body mass index, and high-density lipoprotein cholesterol in a Hispanic-American population. Metab. Syndr. Relat. Disord..

[36] Chiefari E, Tanyolaç S, Iiritano S, Sciacqua A, Capula C, Arcidiacono B, Nocera A, Possidente K, Baudi F, Ventura V, Brunetti G, Brunetti FS, Vero R, Maio R, Greco M, Pavia M, Hodoglugil U, Durlach V, Pullinger CR, Goldfine ID, Perticone F, Foti D, Brunetti A (2013). A polymorphism of HMGA1 is associated with increased risk of metabolic syndrome and related components. Sci. Rep..

[37] Bianco A, Chiefari E, Nobile CGA, Foti DP, Pavia M, Brunetti A (2015). The association between HMGA1 rs146052672 variant and type 2 diabetes: a transethnic meta-analysis. PLoS One.

[38] Chiefari E, Iiritano S, Paonessa F, Le Pera I, Arcidiacono B, Filocamo M, Foti D, Liebhaber SA, Brunetti A (2010). Pseudogene-mediated posttranscriptional silencing of HMGA1 can result in insulin resistance and type 2 diabetes. Nat. Commun..

[39] Arce-Cerezo A, García M, Rodríguez-Nuevo A, Crosa-Bonell M, Enguix N, Peró A, Muñoz S, Roca C, Ramos D, Franckhauser S, Elias I, Ferre T, Pujol A, Ruberte J, Villena JA, Bosch F, Riu E (2015). HMGA1 overexpression in adipose tissue impairs adipogenesis and prevents diet-induced obesity and insulin resistance. Sci. Rep..

[40] Brunetti A, Brunetti L, Foti D, Accili D, Goldfine ID (1996). Human diabetes associated with defects in nuclear regulatory proteins for the insulin receptor gene. J. Clin. Invest..

[41] Chiefari E, Arcidiacono B, Possidente K, Iiritano S, Ventura V, Pandolfo R, Brunetti FS, Greco M, Foti D, Brunetti A (2013). Transcriptional regulation of the HMGA1 gene by octamer-binding proteins Oct-1 and Oct-2. PLoS One.

[42] Messineo S, Laria AE, Arcidiacono B, Chiefari E, Luque Huertas RM, Foti DP, Brunetti A (2016). Cooperation between HMGA1 and HIF-1 contributes to hypoxia-induced VEGF and Visfatin gene expression in 3T3-L1 adipocytes. Front. Endocrinol..

[43] Costa V, Foti D, Paonessa F, Chiefari E, Palaia L, Brunetti G, Gulletta E, Fusco A, Brunetti A (2008). The insulin receptor: a new anticancer target for peroxisome proliferator-activated receptor-γ (PPARγ) and thiazolidinedione-PPARγ agonists. Endocr. Relat. Cancer.

[44] Gerrish K, Cissell MA, Stein R (2001). The role of hepatic nuclear factor 1a and PDX-1 in transcriptional regulation of the pdx-1 gene. J. Biol. Chem..

[45] Desvergne B, Michalik L, Wahli W (2006). Transcriptional regulation of metabolism. Physiol. Rev..

[46] Czech MP (2003). Insulin’s expanding control of forkheads. Proc. Natl. Acad. Sci. USA.

[47] Puig O, Tjian R (2005). Transcriptional feedback control of insulin receptor by dFOXO/FOXO1. Genes Dev..

[48] Chiefari E, Ventura V, Capula C, Randazzo G, Scorcia V, Fedele M, Arcidiacono B, Nevolo MT, Bilotta FL, Vitiello M, Palmieri C, Gulletta E, Fusco A, Foti D, Vero R, Brunetti A (2016). A polymorphism of HMGA1 protects against proliferative diabetic retinopathy by impairing HMGA1-induced VEGFA expression. Sci Rep..

[49] Ponugoti B, Dong G, Graves DT (2012). Role of forkhead transcription factors in diabetes-induced oxidative stress. Exp. Diabetes Res..

[50] Behl Y, Krothapalli P, Desta T, Roy S, Graves DT (2009). FOXO1 plays an important role in enhanced microvascular cell apoptosis and microvascular cell loss in type 1 and type 2 diabetic rats. Diabetes.

